# Endobronchial coil lung volume reduction performed on patients with emphysema dominant COPD: Long term follow-up results

**DOI:** 10.5578/tt.202401834

**Published:** 2024-03-26

**Authors:** Kerem ENSARİOĞLU, Bahar KURT

**Affiliations:** 1 Clinic of Pulmonary Diseases, Ankara Atatürk Sanatorium Training and Research Hospital, Ankara, Türkiye; 2 Clinic of Pulmonary Diseases, Ankara Etlik State Hospital, Ankara, Türkiye

## Abstract

**ABSTRACT**

**
Endobronchial coil lung volume reduction performed on patients with emphysema dominant COPD: Long term follow-up results
**

**Introduction:** *Chronic obstructive pulmonary disease (COPD) is a commonly seen, preventable, and treatable disease with permanent respiratory symp- toms and air entrapment that is caused by particle exposure. In case of limited response to traditional treatment protocols, lung volume reduction may be performed in patients with emphysema dominant patterns. In this study, long term follow-up results of the patients who had been operated on by minimal invasive bronchoscopic lung volume reduction surgery by coil placement were reported.
*

**Materials and Methods:** *Records of the patients operated on by coil place- ment were retrospectively investigated, and pulmonary function test (PFT), echocardiography (ECHO), six-minute walking test (6MWT), tomography images, ventilation scintigraphy, and clinical summaries were evaluated. Out of 34 initial candidates, 18 patients were included in the study. Wilcoxon signed-rank test and Spearman’s rho were utilized to compare interventions and follow-up testing.
*

**Results:** *The average age of 18 patients was 62 (50-74) years, and except for one patient, all were males (n= 17). Fifteen patients were operated bilaterally, and the rest were unilaterally operated, with an average of 10 coils placed per coil placement. An average of 90 days was between bronchoscopic coil place- ment, with a follow-up duration of 45 days in between. Mean total follow-up duration was 794 (± 424) days. Pneumonia and pneumonitis were seen in 33% of patients within the first month. Mortality from respiratory causes was found to be 11%, while mortality from all causes was found to be 22%. Statistical difference was observed regarding 6MWT after bronchoscopic vol- ume reduction when compared the initial preoperative values. However, this difference was later lost statistically at the second follow-up performed after
*

*
the completion of both sides. A benefit in improved resting saturation was
*

*
observed after the second procedure, which was not evident after unilateral
*

*
intervention. However, similiar to 6MWT, this benefit was lost at the second follow-up, with resting saturation instead being effected negatively. No difference was observed in PFT results; however, a correlation was seen between FEV1 and walking distance. No specific correlation had been seen in the ECHO evaluation.
*

**Conclusion:** *Benefits regarding 6MWT and resting saturation were observed in patients undergoing minimal invasive bronchoscopic lung volume reduction surgery with coils. This benefit was evident in the short term but was lost as the follow-up duration increased. A relatively high morbidity and mortality rate was also present, further stating the risky nature of pulmonary intervention, even mini- mally invasive procedures, on patients with COPD.
*

**Key words:** *Chronic obstructive pulmonary disease; bronchoscopy; emphysema; bronchoscopic lung volume reduction
*

**ÖZ**

**
Amfizem dominant KOAH’lı hastalarda uygulanan endobronşiyal sarmal akciğer hacim azaltma: Uzun dönem takip sonuçları
**

**Giriş:** *Kronik obstrüktif akciğer hastalığı (KOAH) sık rastlanan, engellenebilen ve tedavi edilebilen; kalıcı respiratuvar semptomlar ve hava hapsiyle görülen ve partikül maruziyeti sebebiyle gelişen bir hastalıktır. Standart tedavi protokollerine yanıtın kısıtlı olduğu durumlarda, amfizem dominant hastalarda akciğer volüm azaltıcı cerrahi ve girişimler yapılabilir. Bu çalışmada sarmal tel ile yapılan minimal invaziv bronkoskopik akciğer hacim küçültücü girişim yapılan hastaların uzun süreli takip sonuçları değerlendirilmiştir.
*

**Materyal ve Metod:** *Sarmal tel yerleşimi yapılan hastaların kayıtları retrospektif olarak incelendi ve solunum fonksiyon testleri (SFT), ekokardiyografi (EKO), altı dakika yürüme testi (6DYT), tomografi görüntülemeleri, ventilasyon sintigrafisi ve klinik değerlendirme notları kayıt altında alındı. Değerlendirmeye alınan 34 hastadan, 18’i çalışmaya dahil edildi. Pre-operatif sonuçlarla takip sonuçları arasındaki korelasyon değerlendirilmesi için Wilcoxon testi ve Spearman rho korelasyon testleri kullanıldı.
*

**Bulgular:** *Çalışmaya dahil edilen 18 hastanın ortalama yaşı 62 (50-74) idi ve bir hasta hariç, erkek idi (n= 17). On beş hastaya bila- teral girişim yapılmıştı ve geri kalanı unilateral idi. İşlem başı ortalama 10 sarmal tel kullanıldı. Bilateral girişimlerde iki işlem arasında ortalama süre 90 gündü ve ortalama takip süresi 45 gün olarak izlenildi. Total takip süresi ortalama 794 (± 424) gün idi. Pnömoni ve pnömonitis ilk ay içinde hastaların %33’ünde görüldü. Respiratuvar sebeplerden kaynaklanan mortalite %11 iken, tüm sebeplerden kaynaklanılan mortalite %22 olarak görüldü. İşlem sonrasında 6DYT’de anlamlı istatistiksel fark izlenildi. Ancak her iki tarafın da tamamlanmasından sonra yapılan ikinci kontrolde bu fark istatistiksel olarak kayboldu. İkinci işlemden sonra istirahat satürasyonunda iyileşme gözlendi ancak bu tek taraflı bronkoskopik sarmal tel yerleştirilmesi sonrasında belirgin değildi. 6DYT’ye benzer şekilde bu fayda ikinci kontrolde kaybolmuştu ve istirahat satürasyonu olumsuz yönde etkilenmişti. SFT sonuçlarında anlamlı bir farklılık görül-
*

*
mezken FEV1 ve yürüme mesafesi arasında pozitif korelasyon vardı. EKO bulguları ile diğer parametreler arasında anlamlı bir korelas- yon görülmedi.
*

**Sonuç:** *Sarmal tel ile yapılan minimal invazif bronkoskopik akciğer hacim küçültücü girişimlerinde hastalarda 6DYT ve istirahat satü- rasyonu ile ilgili faydalar gözlendi. Bu fayda kısa vadede belirgindi ancak takip süresi arttıkça kayboldu. Yüksek bir morbidite ve mortalite oran olan bu girişim, KOAH’lı hastalarda pulmoner müdahalenin, minimal invaziv prosedürlerin dahi riskli olduğunu gös- termektedir.
*

**Anahtar kelimeler:** *Amfizem; bronkoskopi; bronkoskopik hacim küçültücü girişim; kronik obstruktif akciğer hastalığı
*

## INTRODUCTION

Chronic obstructive pulmonary disease (COPD) is a common, preventable cause of disability that is caused by inhalation of smoke and particles, which in turn create respiratory symptoms and air entrapment (1). Formerly staged by the results of pulmonary function tests (PFT), the current recommendation of the global initiative for chronic obstructive lung disease (GOLD) includes an investigation of symptoms by the COPD Assessment Test (CAT) and/or modified medical research council (mMRC) and hospitalization history for COPD to stage a patient properly (1).

It is well known that COPD, especially at later stages, severely diminishes a patient’s daily activities (2). Traditionally, two components cause COPD’s symptoms: chronic bronchitis, a clinical diagnosis and emphysema, and a pathological change in distal

airways and alveolar units (3,4). Treatment modalities are often presented according to the patient’s GOLD stage, which consists of smoking cessation, pulmonary rehabilitation, inhalation of beta-agonists and leutrine agonists, steroids, and oxygenation support (5,6).

For patients classified as GOLD stage who do not benefit from maximum treatment, alternative treatment modalities include medical approaches, such as roflumilast or long-term prophylactic antibiotics, and surgical approaches to reduce emphysema volume (7-9). Among lung volume reduction surgery (LVRS) methods, a minimally invasive bronchoscopic approach with the implantation of endobronchial coils has been evaluated extensively (10-13).

Generally, the benefit of endobronchial coils has been reported in the mentioned studies, with the

univocally stated benefit being an overall increase in the six-minute walking test (6MWT) or an increase in similar effort evaluation methods. As the progressive nature of COPD is evident, this study aimed to investigate the long-term effect of endobronchial coils on 6MWT and PFT.

### MATERIALS and METHODS

The study was performed as a single-center, retrospective study at a tertiary care hospital’s pulmonary medicine clinic. The patients’ data were collected from the hospital’s computed medical records system, and in cases where the system was not deemed adequate, manual records of the patients were retrieved from the archive files. This was especially the preferred method for evaluating pulmonary function tests (PFT).

Patients who had been operated on for coil insertion by flexible bronchoscopy at the hospital between the years of 2015 and 2020 were accepted as the study population. For coil volume reduction intervention, the patients had to satisfy the inclusion criteria, which were mostly correlated with COPD diagnosis, limited response to traditional treatment, and lack of severe extrapulmonary comorbidity that may limit exercise capacity (Table 1).

Per the pulmonary medicine clinic’s protocol, patients’ evaluation results were recorded in the hospital computed patient chart and the patients’ files for archive purposes. The same procedure was used for follow-up results and comorbidities. The primary initial and follow-up evaluation parameters were PFT, echocardiography, tomography results, and functional performance tests (6MWT). The patients had been on a follow-up regimen, with one follow- up evaluation after each operated side within 45 days, and if considered stable, a follow-up every three months.

The pulmonary medicine clinic had a specific follow- up methodology for patients undergoing coil volume reduction, which included PFT, echocardiography, and 6MWT, as stated above. Patients’ PFT was performed post-bronchodilator therapy to provide a reliable comparison, as all patients had been on bronchodilator therapy consisting of high-dosage corticosteroids, long-term muscarinic agonists, and beta-agonists. 6MWTs were performed upon the initial evaluation and during the follow-up period, with oxygen saturation by finger probe, systolic and diastolic blood pressure, and pulse rate being recorded in addition to distance walking and time spent (in cases where a patient could not endure the six minutes duration). If present, desaturation was reported by a percentage compared to the saturation at the beginning of the test. To exclude cardiac pathologies and to investigate newly occurred ones, echocardiography was performed before each procedure and at the follow-up performed after both sides were operated on.

### Statistical Evaluation

IBM Statistical Package for Social Sciences (SPSS) v22 for Windows was utilized for statistical evaluation. Due to low counts of parameters and patients, non- parametric evaluation was preferred. The Wilcoxon Sign test was used to compare two related groups. Similarly, Kruskal Wallis-H was utilized to compare more than two groups, and Spearman’s correlation was chosen to investigate the correlation between two variables. A p-score lower than 0.05 was accepted as statistically significant. When appropriate, all values were given as average, mean, median, and standard deviation (SD).

The design of this study, its presentation as a doctoral thesis, and the study itself were approved by the local ethics committee. All patients had given written and

verbal informed consent for the intervention and the study, which entailed follow-up evaluation.

## RESULTS

Thirty-four patients were included in the study and evaluated for coil lung volume reduction intervention. Sixteen patients were not considered acceptable for the procedure due to varying comorbidities and contraindications, with diffuse emphysema (n= 4) and pulmonary nodules without adequate follow-up period (n= 3) being the most common observed exclusion criteria (Figure 1). The remaining 18 patients had been deemed suitable for the procedure, with 15 patients being operated bilaterally. An average of 10 coils were inserted per intervention, with the right lung being the first operated side. Median age of the patients was 63.5 (54.5-69) years. The group was predominantly male (n= 17), with one female patient present (Table 2).

Three patients with the unilateral bronchoscopic coil placement were not operated on again due to initiation of smoking, progression to bullous lung,

and organ failure due to urosepsis, respectively. Ventilation scintigraphy was used to evaluate the role of the upper lobes in respiratory capacity, in which the right and left upper lobes were responsible for 8.5% (5.5-12) and 11% (7-15) of the total lung capacity, respectively. Bilaterally operated patients had an average duration of 90 days (± 10) between procedures and had a control follow-up 45 days (± 5) after each performed procedure. After the end of the second follow-up, the remaining follow-up evaluations were performed within three-month intervals up to one year, and then the duration was changed to six monthly evaluations. Mean follow-up duration was 751 (511-1194) days for all patients, and when the patients lost during follow-up due to exitus were excluded from the analysis, the duration was observed to be 831.5 (625-1235) days (Table 2).

Complications that could be related to the procedure were observed at a 33% rate within the first month, with pneumonia and pneumonitis being the most common observed complications. All patients responded well to the treatment, with macrolides and

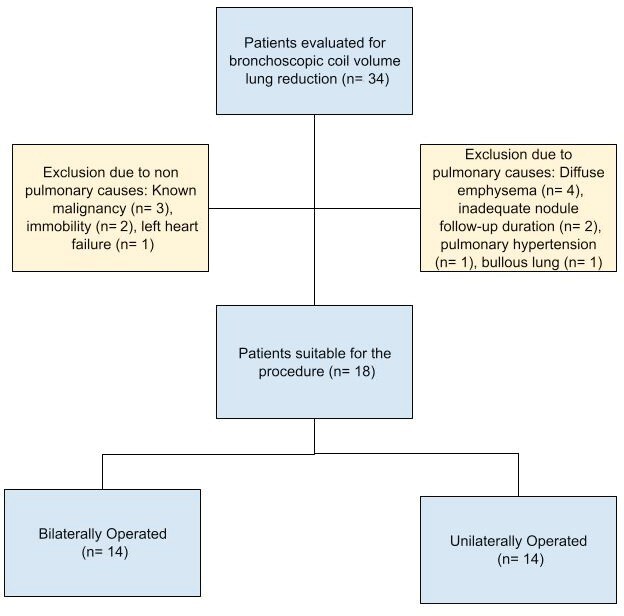

**Figure 1.** Patient evaluation flow chart.

**Table:** 

**Table 2.** Demographic information, procedure evaluation, follow-up and complications		
**Demographic Information**	**Parameters**	
Sex (n,%)	Male Female	17 (94.4) 1 (5.6)
Age (median, 25-75th)		63.5 (54.5-69)
Coils inserted (%, SD)	First procedure Second procedure	10.16 (± 0.85) 9.71 (± 0.99)
	Right upper	8.5 (5.5-12)
	Right middle	25 (23-29)
Ventilation scintigraphy lung area contribution (median, 25-75th)	Right lower Left upper	15 (12.5-18.5) 11 (7-15)
	Left middle	24 (20.5-28)
	Left lower	14 (8.5-16)
Follow-up duration (days) (median, 25-75th)	All patients Exitus excluded	751 (511-1194) 831.5 (625-1235)
	Pneumonia	6 (33.3%)
	Pneumonitis	2 (11%)
Procedure related complications (n, %)	Chest pain	6 (33.3%)
	Dyspnea	15 (84%)
	Hemoptysis	15 (84%)
Exitus	Respiratory-related Other causes	2 (11%) 4 (22%)
SD: Standart deviation, Exitus excluded: This definition included those who were not lost to follow-up.

cephalosporin combinations being utilized for pneumonia and; macrolide and glucocorticoid regimens for pneumonitis. In the mortality analysis, one patient was lost at the second bronchoscopic volume reduction due to an ST-elevated myocardial infarct. One patient was later lost due to respiratory failure after the end of the second post-operative evaluation. An all-cause mortality of 22% (n= 4) was observed, with 11% (n= 2) being attributed to respiratory causes (Table 2). Kaplan-Meier survival analysis showed an estimated survival of 1327.6 days, with a range of 1057.4-1597.8 days (Figure 2).

The most commonly observed complaint attributed to the procedure was chest pain (n= 6, 33.3%), which was pleuritic and occurred within the first week. Hemoptysis and dyspnea were commonly observed in the initial post operative day in most patients (84%). Hemoptysis disappeared on all patients on the second day, with dyspnea often lasting until discharge, with all cases being relieved within the first week. No long-term complication was reported after the first month of intervention (Table 2).

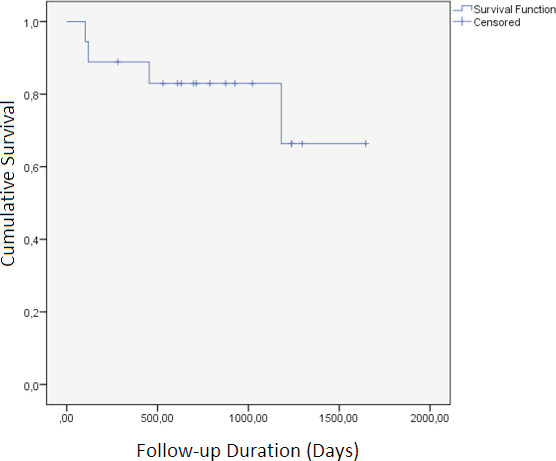

**Figure 2.** Survival analysis.

Comparisons were made between the parameters by the Wilcoxon Sign test, which compared initial testing results, the second evaluation performed before the bilateral procedure, after the procedure was performed on both sides, and the first follow-up

**Table:** 

**Table 3.** Wilcoxon Sign comparison between the first procedure and first follow-up				
**Parameters**		**Median (25-75th)**	**p**	**Z**
**First Procedure** **First Follow-up**	Resting saturation (%)	88 (85-92.5) 91.5 (87.5-93.5)	0.247	-1.158
**First Procedure** **First Follow-up**	Desaturation ratio (%)	5 (1-13.5) 6 (0.5-14.25)	0.423	-.801
**First Procedure** **First Follow-up**	Resting pulse rate (/minute)	97 (83.5-119.5) 99 (65.75-105.25)	0.727	-.350
**First Procedure** **First Follow-up**	Change in pulse rate (%)	11 (4-24) 17(9-30.25)	0.780	-.280
**First Procedure** **First Follow-up**	Walked distance (m)	120 (90-340) 227.5 (180-375)	**0.004**	-2.905
**First Procedure** **First Follow-up**	Walking duration (s)	150 (120-360) 360 (243.75-360)	**0.021**	-2.312
**First Procedure** **First Follow-up**	Mean arterial pressure (mmHg)	97 (87-104) 88.5 (77-102.25)	0.196	-1.294
**First Procedure** **First Follow-up**	Change in MAP	7 (3-18.5) 12 (0.75-23)	0.196	-1.294
**First Follow-up** **First Procedure**	Forced expiratory volume 1 (%)	34 (22-40) 30.5 (24.5-38.25)	0.918	-.102
**First Follow-up** **First Procedure**	Forced vital capacity (%)	54 (40-65) 54.5 (42.5-66)	0.306	-1.023
**First Follow-up** **First Procedure**	DLCO (%)	51 (26.75-78.5) 54 (29-74)	0.854	-.184
**First Follow-up** **First Procedure**	Total lung capacity (%)	111 (101.25-143) 124 (121-140)	0.180	-1.342
**First Follow-up** **First Procedure**	Residual volume (%)	196 (170.5-288.75) 229.00 (198-290.5)	0.180	-1.342
6MWT: 6 minutes walking test, MAP: Mean arterial pressure, DLCO: Diffusing capacity for carbon monoxide. SD: Standart deviation. Desaturation Ratio was calculated by the ratio of finger probe saturation percentage at the end of 6MWT result, divided by the initial ratio. Mean arterial pressure was calculated using non-invasive methods.

performed afterward. There was a significant difference in walking distance between initial testing performed before the first procedure and the first follow-up (a median of 120 meters to 227.5 meters, respectively, p= 0.004). Walking duration also increased, with four patients already at the six- minute mark and retaining that duration and seven patients having an increased walking duration (p= 0.021). No significant difference was present for other parameters (Table 3).

A similar difference in walking distance was present after the second procedure, with a median walking distance of 277.5 meters (p= 0.041), an overall

increase of 25 meters compared to the first. Resting saturation also improved in the patients, which was not observed after the first procedure (a median of 88% to 92.5%, p= 0.035) (Table 4). At the second follow-up after completing both procedures (third overall evaluation), no statistically significant benefit was observed, with resting saturation being affected negatively (p= 0.005). The distance benefit was still present; however, it had lost its statistical significance (a median distance of 120 meters to 207.5, p= 0.575) (Table 5). No significant difference was present regarding ECHO findings during follow-up period (Table 6).

**Table:** 

**Table 4.** Wilcoxon Sign comparison between the first procedure and second follow-up				
**Parameters**		**Median (25-75th)**	**p**	**Z**
**First Procedure** **Second Follow-up**	Resting saturation (%)	88 (85-92.5) 92.5 (88-94.5)	**0.035**	-.140
**First Procedure** **Second Follow-up**	Desaturation ratio (%)	5 (1-13.5) 4 (0-11.75)	0.836	-2.040
**First Procedure** **Second Follow-up**	Resting pulse rate (/minute)	97 (83.5-119.5) 101 (79.5-109.75)	0.116	-.943
**First Procedure** **Second Follow-up**	Change in pulse rate (%)	11 (4-24) 16 (7.25-24.25)	0.889	-.785
**First Procedure** **Second Follow-up**	Walked distance (m)	120 (90-340) 277.5 (120-420)	**0.041**	-.175
**First Procedure** **Second Follow-up**	Walking duration (s)	150 (120-360) 321 (172.5-360)	0.345	-.805
**First Procedure** **Second Follow-up**	Mean arterial pressure (mmHg)	97 (87-104) 91.5 (86-97.75)	0.433	-.910
**First Procedure** **Second Follow-up**	Change in MAP	7 (3-18.5) 8 (2.25-25.25)	0.861	-1.000
**First Procedure** **Second Follow-up**	Forced expiratory volume 1 (%)	34 (22-40) 35 (25.25-40.25)	0.421	-1.342
**First Procedure** **Second Follow-up**	Forced vital capacity (%)	54 (40-65) 63 (46.5-70.75)	0.363	-1.342
**First Procedure** **Second Follow-up**	DLCO (%)	51 (26.75-78.5) 31 (27.5-41.25)	0.317	-.105
**First Procedure** **Second Follow-up**	Total lung capacity (%)	111 (101.25-143) 105.5 (99.75-145)	0.180	-.944
**First Procedure** **Second Follow-up**	Residual volume (%)	196 (170.5-288.75) 194.00	0.180	-.944
6MWT: 6 minutes walking test, MAP: Mean arterial pressure, DLCO: Diffusing capacity for carbon monoxide, SD: Standart deviation. Desaturation ratio was calculated by the ratio of finger probe saturation percentage at the end of 6MWT result, divided by the initial ratio. Mean arterial pressure was calculated using non-invasive methods.				

## DISCUSSION

Both procedures showed benefit in performance regarding six-minute walking distance, with a walking distance increase of 70-100 meters in each procedure. Compared to the first procedure, the walking distance increase was more prominent in the second procedure, while the walking duration increase was only present after the first procedure. This difference between the initial procedure and the completion of the bilateral procedure justifies that, while unilateral coil insertion is a viable approach, the bilateral procedure is too justified. This justification is further supported by the observation that patients’ resting saturation improved during the follow-up period,

which was not observed after the first procedure. While beneficial after the second procedure, overall benefit observed in general performance reduced as follow-up duration increased, with reduced walking distance and resting saturation levels closer to pre- operative values.

No statistical significance was observed in FEV1 and FVC values regarding respiratory function tests. DLCO, TLC, and RV values also did not change significantly after the procedure and during the follow-up period; however, this was attributed to the low number of patients being able to perform testing for DLCO reliably, thus limiting the available data for comparison.

**Table:** 

**Table 5.** Wilcoxon Sign comparison between the first procedure and third follow-up				
**Parameters**		**Median (25-75th)**	**p**	**Z**
**First Procedure** **Third Follow-up**	Resting saturation (%)	88 (85-92.5) 86 (83-88.25)	**0.005**	-2.820
**First Procedure** **Third Follow-up**	Desaturation ratio (%)	5 (1-13.5) 8 (2-15)	0.462	-.735
**First Procedure** **Third Follow-up**	Resting pulse rate (/minute)	97 (83.5-119.5) 76 (72-104.5)	0.249	-1.153
**First Procedure** **Third Follow-up**	Change in pulse rate (%)	11 (4-24) 36.5 (16.25-54.5)	0.598	-.527
**First Procedure** **Third Follow-up**	Walked distance (m)	120 (90-340) 207.5 (135-322.5)	0.575	-.560
**First Procedure** **Third Follow-up**	Walking duration (s)	150 (120-360) 360 (195-360)	0.225	-1.214
**First Procedure** **Third Follow-up**	Mean arterial pressure (mmHg)	97 (87-104) 93 (82.25-100)	0.686	-.405
**First Procedure** **Third Follow-up**	Change in MAP	7 (3-18.5) 14 (8.25-25.5)	0.753	-.314
**First Procedure** **Third Follow-up**	Forced expiratory volume 1 (%)	34 (22-40) 35 (28-36)	0.952	-.060
**First Procedure** **Third Follow-up**	Forced vital capacity (%)	54 (40-65) 54 (50-73)	0.756	-.311
**First Procedure** **Third Follow-up**	DLCO (%)	51 (26.75-78.5) 39 (23.5-53.5)	0.285	-1.069
6MWT: 6 minutes walking test, MAP: Mean arterial pressure, DLCO: Diffusing capacity for carbon monoxide, SD: Standart deviation. Desaturation ratio was calculated by the ratio of finger probe saturation percentage at the end of 6MWT result, divided by the initial ratio. Mean arterial pressure was calculated using non-invasive methods.				

**Table:** 

**Table 6.** Echocardiography Wilcoxon Sign comparison between the procedures and follow-up evaluations				
**Echocardiograpy Findings**		**Median (25-75th)**	**p**	**z**
**First Procedure**		60 (59.5-60)		
**Second Procedure**		60 (59.25-60)	0.916	-.106
	Ejection fraction (%)			
**Second Follow-up**		60.00 (60-60)	0.414	-.207
**Third Follow-up**		60.00 (60-60)	1.000	.000
**First Procedure**		32.5 (25-40)		
**Second Procedure**		33 (29.5-48.5)	0.916	-0.105
	Systolic arterial pulmonary pressure (mmHg)			
**Second Follow-up**		36 (29-48.25)	0.600	-1.570
**Third Follow-up**		45 (30-58.5)	0.204	1.270
SD: Standart deviation. All p and z values were given with comparison to the first procedure values.

Other studies have shown that FEV1 values had a short-term benefit, with long-term results either being unavailable or having a benefit, yet with limited significance (14,15). As for DLCO testing, it has been

reported that patients with higher pre-operative TLC and RV values had better follow-up results and a longer benefit duration than those with lower values (16,17). A positive correlation between FEV1 and

6MWT distance was observed in the study, which was an expected result. Patients who had increased 6MWT distances also had a positive increase in FEV1 values.

Cardiac evaluation did not reveal a change in sPAB values of echocardiography during follow-up, and no correlation was found between sPAB and other parameters. Two studies have also utilized echocardiography to evaluate patients, regardless of former cardiac history, to exclude cardiac pathologies; however, repeated measurements of sPAB have not been performed before in the literature (12,13,18). Current results in our study support the notion that, while ECHO may be used for initial evaluation, there is not enough data to justify repeated cardiac investigation.

Similar to our study, respiratory-related mortality in the literature review was observed within the first month. Most studies have reported long-term all- cause mortality at 30-40%, while our study reported it at 22% (13,16-18). Compared to the literature, this reduction was attributed to the smaller patient population. The prominent respiratory complication profile of the procedure was an increase in COPD exacerbation and pneumonia. While this increase was observed in our study, the exact ratios in the literature vary, with the study’s results being 33% in terms of pneumonia and literature rates being between 3% and 35% (12,14,19). An interesting outcome was that, unlike other studies, there were no patients with pneumothorax in our group.

To summarize, the procedure was beneficial in the short term regarding 6MWT performance and provided better resting saturation. However, as the follow-up duration increased, the significance of the benefits was lost. With varying mortality and morbidity rates, patients should be notified of expected results and risks beforehand to better assess the acceptability of the procedure compared to traditional care. The transient nature of the procedure, combined with the progressive nature of COPD, may lead to some potential patients forging these methods in favor of other treatment options and palliative support.

Our study varied from the available literature present, as long-term follow-up revealed that, despite having positive effects in the short-term, a long-term benefit

would be eventually lost, and no evident benefit was observed regarding respiratory function test benefits. Additional observation of cardiac performance was made, however, in this investigation no benefit was noted.

The primary limitation of the study was the limited patient count. In addition, as stated in comparison analysis tables, patients had new comorbidities and clinical regression over the long follow-up period, preventing some patients from achieving ideal performance testing. While increasing patient count might have resolved this issue, the fact remains that this procedure was utilized on patients with already limited functional capacity, and thus, even with a larger population, patient functional assessment may still be suboptimal. Another limitation of the study was that an objective evaluation of symptom relief could not be performed, as patients could not reliably fill the Saint George Respiratory Questionnaire (SGRQ). Similarly, most pulmonary function test

comparisons could only be made by FEV1 and FVC values, as cooperation with DLCO testing was

severely limited.

Confounding parameter limitation was partially achieved due to extensive patient selection already provided by the preoperative evaluation, which excluded many comorbidities. Selection bias was another issue encountered, as due to the nature of the study, a control group could not be chosen, however, this was relatively controlled, as the study was mainly performed with evaluations performed with values provided by the same patients over a long duration (interpatient), instead of comparing patients with each other.

## CONCLUSION

Bronchoscopic volume reduction with the coil provides a transient benefit regarding increased walking distance in 6MWT and a higher resting saturation rate. While evident in the initial follow-up after the completion of the bilateral procedure, the walking distance had lost its statistical significance after the second follow-up, and similarly, resting saturation had been affected negatively. A relatively high morbidity and mortality rate was also present, further stating the risky nature of pulmonary intervention, even minimally invasive procedures, on patients with COPD.

**Ethical Committee Approval:** This study was approved by the University of Health Sciences Dışkapı Yıldırım Beyazıt Training and Research Hospital (Decision no: 87/01, Date: 08.05.2020).

### CONFLICT of INTEREST

The authors declare that they have no conflict of interest.

## AUTHORSHIP CONTRIBUTIONS

Concept/Design: KE, BK Analysis/Interpretation: KE, BK Data acqusition: KE, BK Writing: KE, BK

Clinical Revision: BK Final Approval: BK

## REFERENCES

1. *
  Singh D, Agusti A, Anzueto A, Barnes PJ, Bourbeau J, Celli BR, et al. Global strategy for the diagnosis, manage- ment, and prevention of chronic obstructive lung dis- ease: The GOLD science committee report 2019. Eur Respir J 2019; 53(5): 1900164. https://doi. org/10.1183/13993003.00164-2019*
2. *
  Lange P, Marott JL, Vestbo J, Olsen KR, Ingebrigtsen TS, Dahl M, et al. Prediction of the clinical course of chronic obstructive pulmonary disease, using the new GOLD clas- sification: A study of the general population. Am J Respir Crit Care Med 2012; 186(10): 975-81. https://doi. org/10.1164/rccm.201207-1299OC*
3. *
  Elbehairy AF, Raghavan N, Cheng S, Yang L, Webb KA, Neder JA, et al. Physiologic characterization of the chron- ic bronchitis phenotype in GOLD Grade IB COPD. Chest 2015; 147(5): 1235-45. https://doi.org/10.1378/ chest.14-1491*
4. *
  Sharafkhaneh A, Hanania NA, Kim V. Pathogenesis of emphysema: From the bench to the bedside. Proc Am Thorac Soc 2008; 5(4): 475-7. https://doi.org/10.1513/ pats.200708-126ET*
5. *
  Schirnhofer L, Lamprecht B, Vollmer WM, Allison MJ, Studnicka M, Jensen RL, et al. COPD prevalence in Salzburg, Austria: Results from the Burden of Obstructive Lung Disease (BOLD) study. Chest 2007; 131(1): 29-36. https://doi.org/10.1378/chest.06-0365*
6. *
  Bone R, Boyars M, Braun SR, Buist AS, Campbell S, Chick T, et al. In chronic obstructive pulmonary disease, a com- bination of ipratropium and albuterol is more effective than either agent alone: An 85-day multicenter trial. Chest 1994; 105(5): 1411-9. https://doi.org/10.1378/ chest.105.5.1411*
7. *
  Martinez FJ, Calverley PMA, Goehring UM, Brose M, Fabbri LM, Rabe KF. Effect of roflumilast on exacerbations in patients with severe chronic obstructive pulmonary disease uncontrolled by combination therapy (REACT): A multicentre randomised controlled trial. Lancet 2015; 385(9971): 857-66. https://doi.org/10.1016/S0140-*

*6736(14)62410-7*

8. *
  Boasquevisque CHR, Yildirim E, Waddel TK, Keshavjee S. Surgical techniques: Lung transplant and lung volume reduction. Proc Am Thorac Soc 2009; 6(1): 66-78. https://doi.org/10.1513/pats.200808-083GO*
9. *
  DeCamp MM, Lipson D, Krasna M, Minai OA, McKenna RJ, Thomashow BM. The evaluation and preparation of the patient for lung volume reduction surgery. Proc Am Thorac Soc 2008; 5(4): 427-31. https://doi.org/10.1513/ pats.200707-087ET*
10. *
  Gordon M, Duffy S, Criner GJ. Lung volume reduction surgery or bronchoscopic lung volume reduction: Is there an algorithm for allocation? J Thorac Dis 2018; 10(Suppl 23): S2816-S23. https://doi.org/10.21037/ jtd.2018.05.118*
11. *
  Herth FJ, Eberhard R, Gompelmann D, Slebos DJ, Ernst A. Bronchoscopic lung volume reduction with a dedicated coil: A clinical pilot study. Ther Adv Respir Dis 2010; 4(4): 225-31. https://doi.org/10.1177/1753465810368553*
12. *
  Sciurba FC, Criner GJ, Strange C, Shah PL, Michaud G, Connolly TA, et al. Effect of endobronchial coils vs usual care on exercise tolerance in patients with severe emphysema: The renew randomized clinical trial. JAMA 2016; 315(20): 2178-89. https://doi.org/10.1001/ jama.2016.6261*
13. *
  Deslée G, Mal H, Dutau H, Bourdin A, Vergnon JM, Pison C, Kessler R, et al. Lung volume reduction coil treatment vs usual care in patients with severe emphysema the REVOLENS randomized clinical trial. JAMA 2016; 315(2): 175-84. https://doi.org/10.1001/jama.2015.17821*
14. *
  Gülşen A. Bronchoscopic lung volume reduction: A 2018 review and update. Turk Thorac J 2018; 19(3): 141-9. https://doi.org/10.5152/TurkThoracJ.2018.18044*
15. *
  Marchetti N, Kaufman T, Chandra D, Herth FJ, Shah PL, Slebos DJ, et al. Endobronchial coils versus lung volume reduction surgery or medical therapy for treatment of advanced homogenous emphysema. Chronic Obstr Pulm Dis 2018; 5(2): 87-96. https://doi.org/10.15326/ jcopdf.5.2.2017.0134*
16. *
  Venuta F, Anile M, Diso D, Carillo C, De Giacomo T, D’Andrilli A, et al. Long-term follow-up after bronchoscop- ic lung volume reduction in patients with emphysema. Eur Respir J 2012; 39(5): 1084-9. https://doi. org/10.1183/09031936.00071311*
17. *
  Hartman JE, Klooster K, Gortzak K, Ten Hacken NHT, Slebos DJ. Long-term follow-up after bronchoscopic lung volume reduction treatment with coils in patients with severe emphysema. Respirology 2015; 20(2): 319-26. https://doi.org/10.1111/resp.12435*
18. *
  Deslee G, Klooster K, Hetzel M, Stanzel F, Kessler R, Marquette CH et al. Lung volume reduction coil treatment for patients with severe emphysema: A European multi- centre trial. Thorax 2014; 69(11): 980-6. https://doi. org/10.1136/thoraxjnl-2014-205221*
19. *
  Welling JBA, Slebos DJ. Lung volume reduction with endo- bronchial coils for patients with emphysema. J Thorac Dis 2018; 10(Suppl 23): S2797-S805. https://doi. org/10.21037/jtd.2017.12.95*

